# The effect of macromolecular crowding degree on the self-assembly of fatty acid and lipid hydrolysis

**DOI:** 10.1038/s41538-023-00213-2

**Published:** 2023-07-26

**Authors:** Yu-Long Sun, Bing-Qiang Ge, Mi-Zhuan Li, Lei Wang, Zhong-Xiu Chen

**Affiliations:** 1 Molecular Food Science Laboratory, College of Food & Biology Engineering, Zhejiang Gongshang University, Hangzhou, 310018 China; 2 ACON Biotech (Hangzhou) Co., Ltd., Hangzhou, 310018 China

**Keywords:** Fatty acids, Food nanotechnology

## Abstract

Investigation on the physiochemical nature involved in the production of fatty acid catalyzed by the vesicles is of importance to understand the digestion of lipid. In this paper, the effects of crowding degree, which was constructed by polyethylene glycol (PEG), on the autocatalytic production of fatty acid with different chain lengths was studied. The results showed that the higher crowding degree led to the slower production rate of decanoic acid but the faster rate of oleic acid. The reason lies in that the presence of macromolecules resulted in the increased sizes of decanoic acid vesicles, but decreased sizes of oleic acid vesicles. Meanwhile, decanoic acid vesicles in more crowded medium exhibited viscous behavior, whereas oleic acid displayed elastic behavior. This research provides useful information for understanding the unusual autocatalyzed production of fatty acid in macromolecular crowding and may also draw an attention to the physiologically relevant lipid digestion.

## Introduction

Digestion of lipid is the key to the survival of mammal species, yet it is surprising how little we understand this process. The critical region in lipid digestive system is the lipid-water interface, where the key reactions take place to solubilize lipids and lipid soluble nutrients^1^. An early study showed that unilamellar vesicles originated in lamellar liquid crystals that formed at emulsion-water interfaces in the upper small intestine during lipid hydrolysis^2^. During the digestion of dairy milk, normal emulsion transformed into various structures through a variety of ordered nanostructures for maintenance of lipid absorption^3^. These results indicate that understanding the formation of nanostructures, especially the self-assembly of fatty acid and its influence on lipid digestion is of significance for regulating fat digestion and absorption.

Lipid digestion is an enzyme-catalyzed hydrolysis reaction. The digestion of lipid is different from that of other nutrients in that: (1) Lipase is an interface enzyme, and the digestion of lipids is carried out at the oil-water interface. Therefore, the droplet size, interfacial area, surface tension and viscoelasticity affect the digestion efficiency. (2) The digestion of triglycerides is a hydrolysis reaction in which the resulting fatty acids are amphiphilic molecules and important components involved in the formation of nanostructured self-assemblies. Besides, amphiphilic molecules such as phospholipids, cholates, and input emulsifiers were also involved in the self-association process^4^. The self-assemblies of amphiphiles and the evolution of various aggregates inevitably affects the adsorption of surface-active materials on the oil-water interface, and therefore affect the thermodynamic and kinetic processes of lipid digestion. However, the details of the mechanism have not been known yet. How to simplify this complex process and establish a model to explore the relationship between the development and transformation of nanostructured self-assemblies and the efficiency of lipid hydrolysis is a scientific problem that needs to be investigated.

Our preliminary research revealed that autocatalytic hydrolysis of fatty acid anhydrides in crowded medium could provide a model which included hydrolyzed production of fatty acid, vesicle catalysis, and mimic crowded bionetworks^5^. The reason was that hydrolysis of fatty acid anhydrides produced fatty acid, which was a chemically irreversible surface process, including vesicle formation from the generated ionized fatty acid, products distribution between the organic and water phases, solubilization of the hydrophobic compounds by the aggregates^6^. The elements, such as fatty acids produced by hydrolysis, interfacial catalysis, and reactions occurring in confined spaces formed by macromolecules, could describe certain characteristics of lipid digestion in vivo. In fact, vesicles composed of fatty acids has been applied for transdermal delivery of various bioactives^7^. The dynamics of deformations and topological transitions of fatty acid vesicles in response to pH stimuli have been accurately described^8^. However, the association of reaction models involving fatty acid production, vesicle microstructure evolution and lipid digestion containing interfacial catalysis has not been reported. Moreover, the influencing factors of conventional lipid digestion studies usually considered factors such as pH and ionic strength, and few people have paid attention to the factors of macromolecular medium. In view of the fact that a significant fraction of the gut space is occupied by macromolecules, it is important to consider the impact that crowded medium might have on the self-assembly of nanostructures and lipid hydrolysis.

The term “macromolecular crowding” was coined to connote the influence of mutual volume exclusion on the thermodynamic, kinetic and structural properties of macromolecules in crowded medium^9^. It is necessary to measure the quantitative effects of crowding in all studies of macromolecular interactions if these studies were to be regarded as physiologically relevant^10^. We have found that crowded medium constructed by polysaccharides affected the activity of lipase, which in turn regulated lipid digestion^11^. However, the quantitative study on the effect of crowding degree of macromolecular networks on fatty acid-involved self-assembly and lipid digestion remains unclear. In this paper, the changes in the nanostructure of fatty acids and lipid hydrolysis reactions in confined spaces was studied by constructing a simple model of lipid hydrolysis using fatty acid anhydrides as the starting materials. Polyethylene glycol (PEG) with varied molecular weight was selected to construct the crowded medium with adjustable crowding degree. The differences of fatty acids of different chain lengths during the evolution of nanostructures and the mechanisms of their effects on lipid digestion were studied in detail. The research will draw a wide range of attention to the topics of physiochemical events including self-assembly, autocatalytic reaction and macromolecular crowding effect coexisted in food digestion system, which would allow manipulation of physicochemical and interfacial properties to modulate lipid digestion.

## Results and discussion

### Quantitative effects of macromolecular crowding on the rate of autocatalytic production of fatty acid with varied hydrophobic chain length

**Fig. 1:**
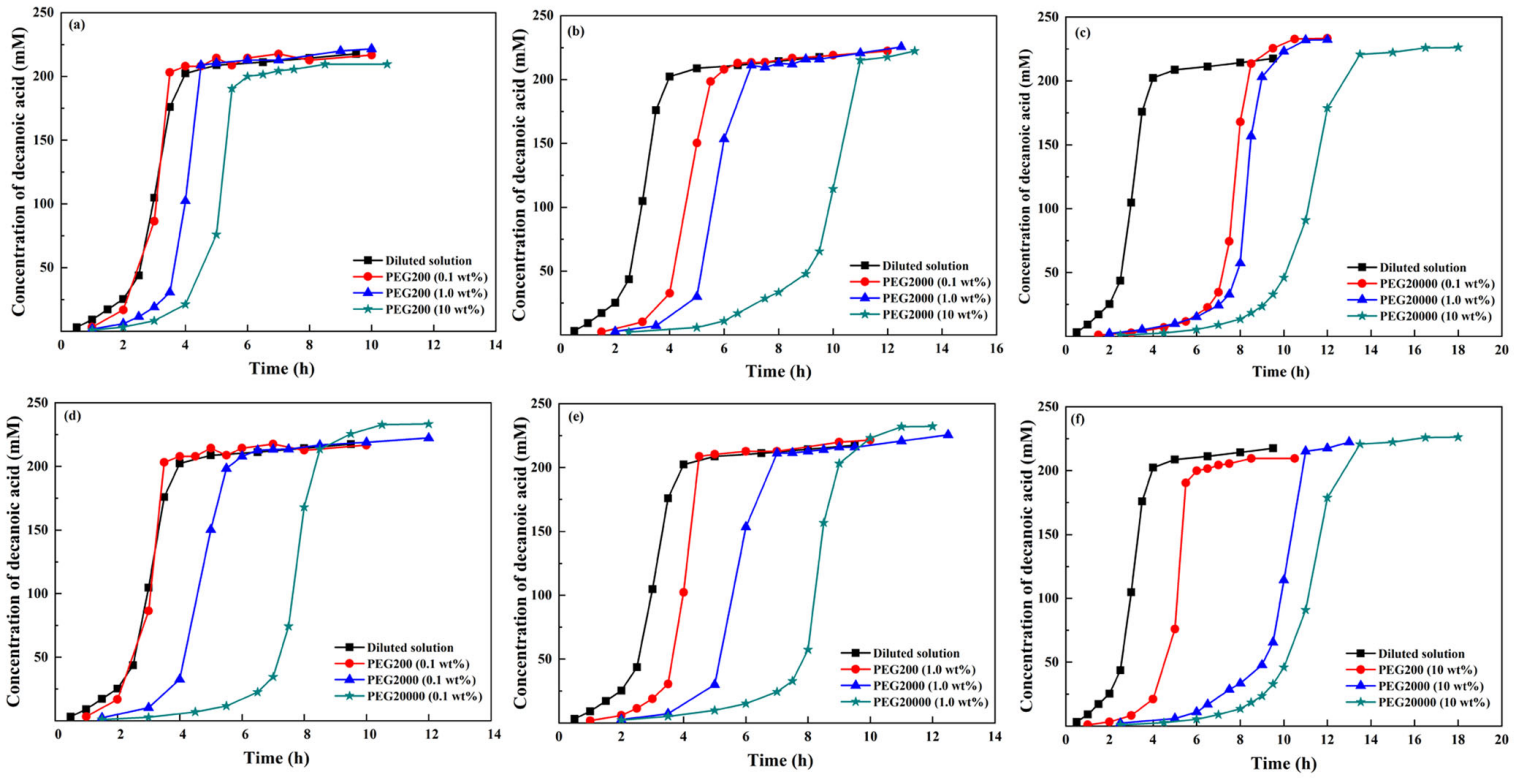
Vesicle-catalyzed production curve of decanoic acid by the hydrolysis of decanoic anhydride in dilute solution and in 0.1 wt%, 1.0 wt%, and 10.0 wt% of PEG200, PEG2000, and PEG20000, respectively. **a**, **b** and **c** showed the autocatalytic hydrolysis curves of decanoic anhydride in dilute solution and in PEG at different concentration and molecular weight. **d**, **e** and **f** were displayed for comparison. Decanoic anhydride was hydrolyzed at 60 ^o^C.

PEG has good biocompatibility which makes it appropriate for mimicking physiological crowding^12^. PEG200, PEG2000, and PEG20000 with adjustable crowding degree was used. Decanoic (C10) acid anhydride and oleic (C18) anhydride were chosen as the typical fatty acid anhydrides. Figure 1 shows the time courses of the vesicle-catalyzed decanoic acid formation in dilute solution and in crowded medium. When the concentration and molecular weight of PEG increased, both the induction time (the lag time) and the time for reaction to complete became longer. In general, the rate of producing C10 fatty acid decreased strongly with an increasing crowding degree.

**Fig. 2:**
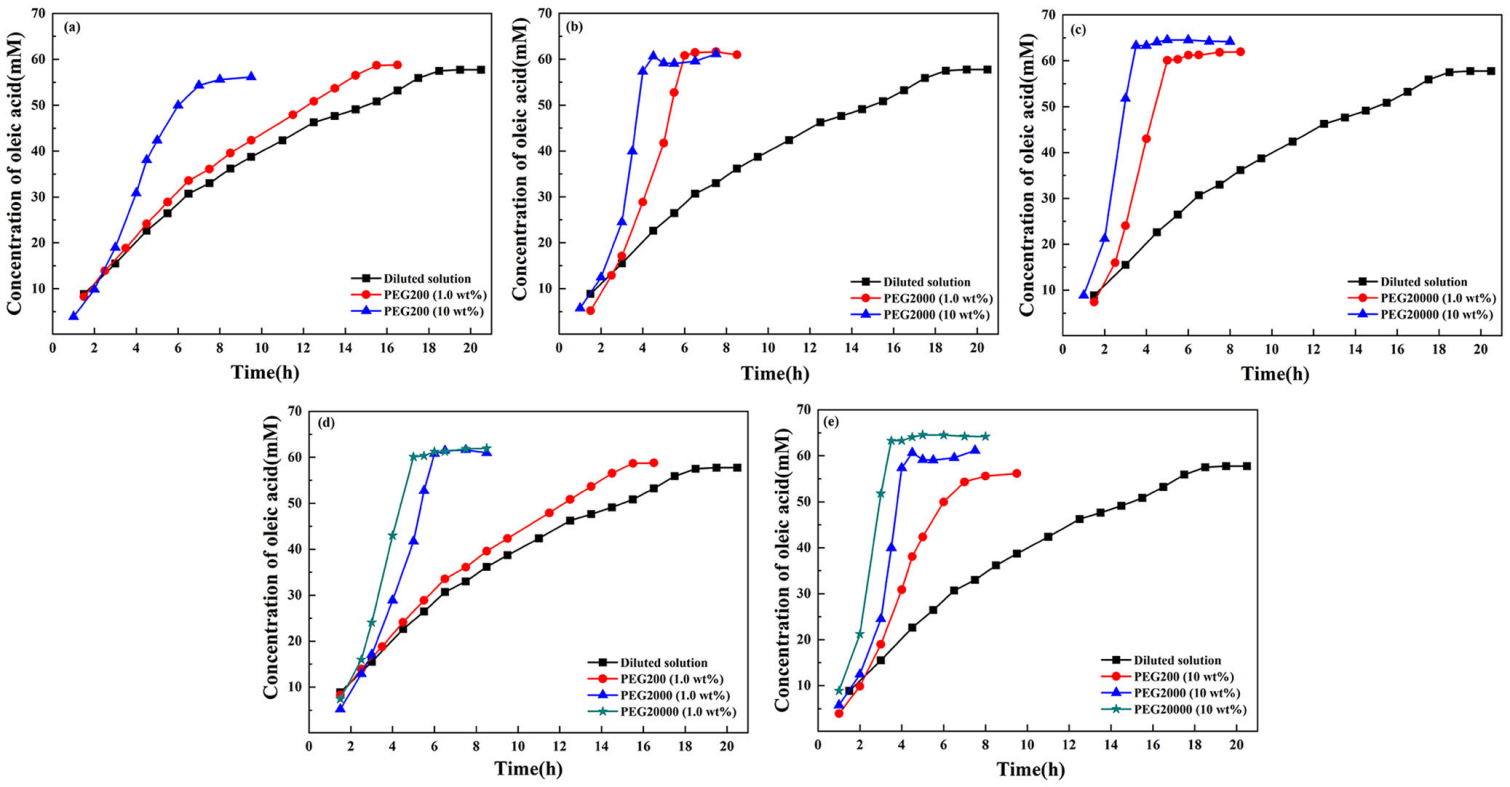
Vesicle-catalyzed production curve of oleic acid by the hydrolysis of oleic anhydride in dilute solution and in 1.0 wt%, and 10.0 wt% PEG200, PEG2000, and PEG20000, respectively. **a**, **b** and **c** showed the autocatalytic hydrolysis curves of oleic anhydride in dilute solution and in PEG at different concentration and molecular weight. **d** and **e** were displayed for comparison. Oleic anhydride was hydrolyzed at 41 ^o^C.

In the case of oleic acid (Fig. 2) whose production could be accelerated by crowding agent, increased crowding degree made the reaction progress faster. Compared with dilute solutions, the presence of crowding agent slowed down the production rate of C10 fatty acid. However, PEG led to increased rate of C18 fatty acid, suggesting that the effects of the crowding on the vesicle-catalyzed production of fatty acid depended on the hydrophobic chain length.

### Investigation on the mechanism behind the unusual effect of macromolecular crowding on the rate of autocatalytic production of fatty acid

#### Characterization of the size, polydispersity and morphology of vesicles formed from the hydrolyzed fatty acid in crowding

**Fig. 3:**
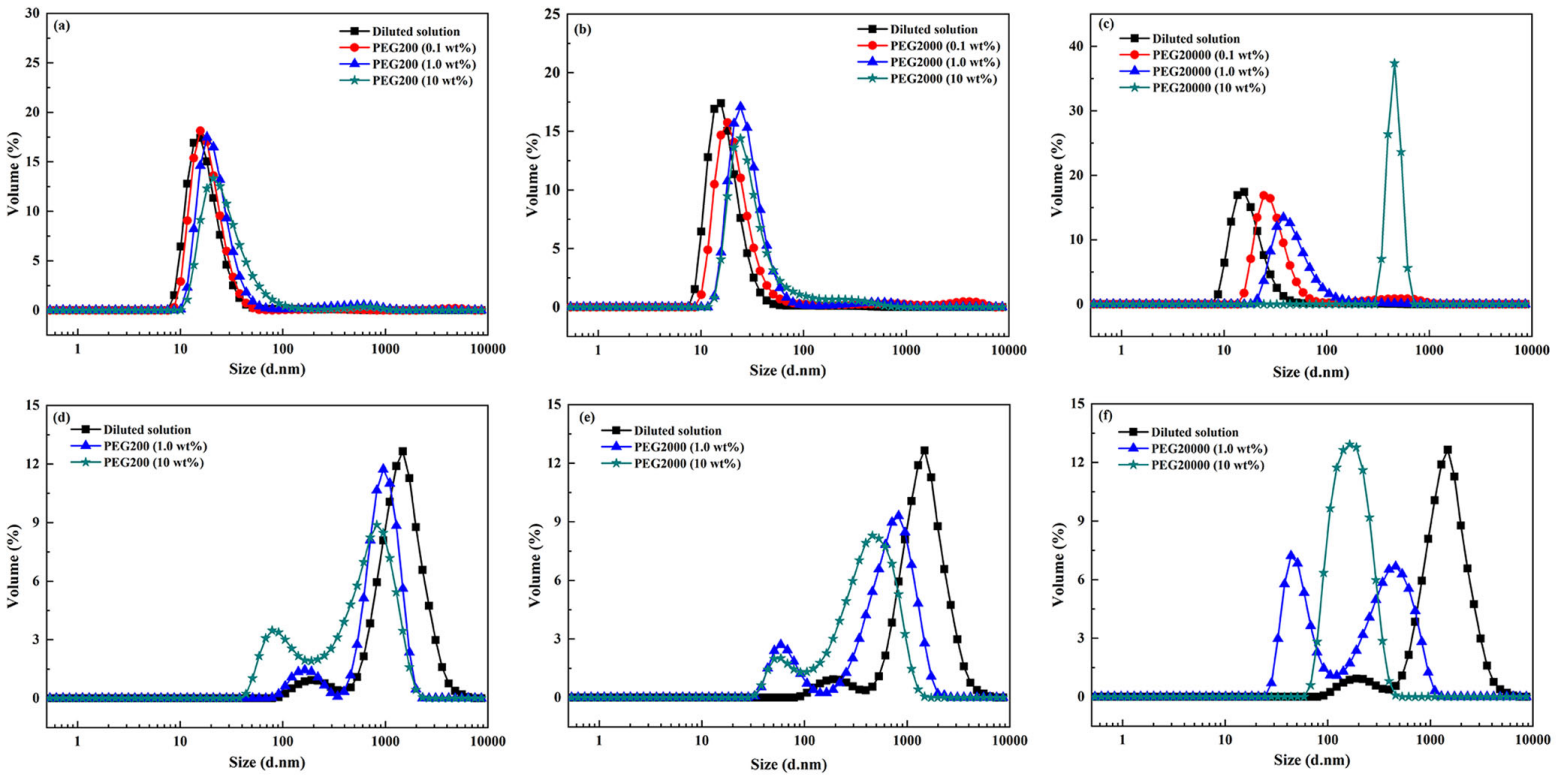
Size distribution of the aggregates during the hydrolysis of fatty acid anhydrides in the presence of PEG with different molecular weights and concentrations. **a**–**c** For decanoic anhydride and **d**–**f** for oleic anhydride. The temperature was 60 °C for decanoic anhydride and 41 °C for oleic anhydride, respectively.

To investigate the role of vesicles on the hydrolysis, the effect of PEG on the size distribution of vesicles were studied. As shown in Fig. 3, vesicles formed during the hydrolysis whether in PEG solution or in dilute buffer. DLS curves for C10 fatty acid vesicles yielded a single peak, indicating a narrow distribution (Fig. 3a–c). In Fig. 3d–f, the vesicles of C18 in dilute buffer and in crowding (except for 10 wt% of PEG20000) had two kinds of particle distribution, and most of the aggregates were larger than that of C10 vesicles. For C10 fatty acid, increased crowing resulted in the formation of large aggregates. However, in the case of C18 fatty acid, smaller aggregates formed, suggesting that the effect of crowded medium on the vesicle size was unusually chain-length dependent. The increased C10 vesicle size probably came from the similar depletion force^13^. But for C18 vesicles, different interaction might be involved.

**Fig. 4:**
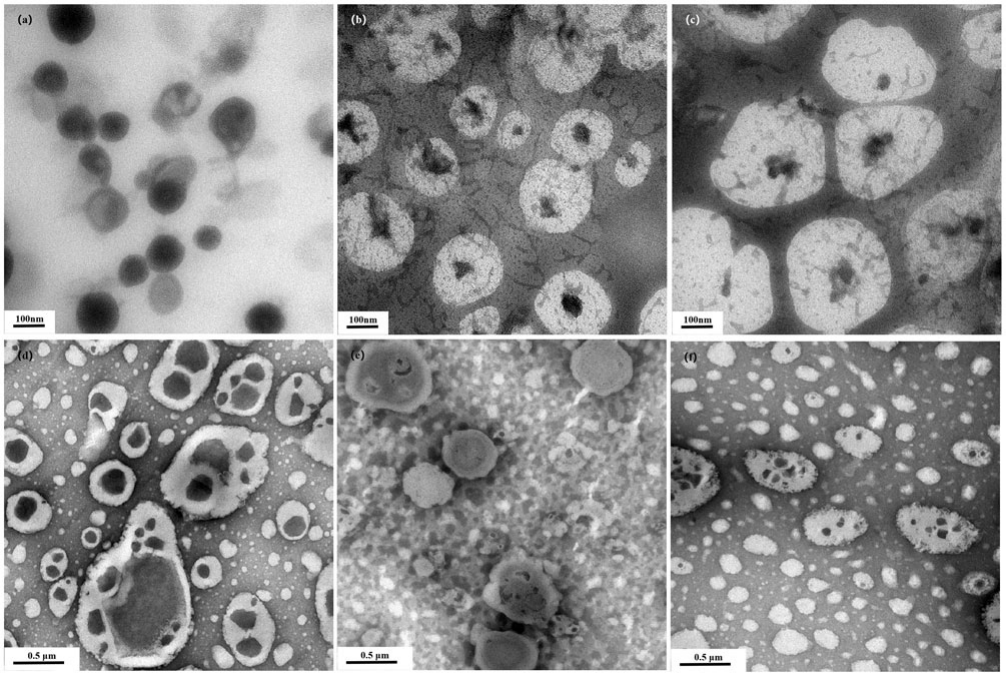
Transmission electron micrographs (TEM) of fatty acid vesilcels. **a**–**c** For decanoic acid/decanoate vesicles and **d**–**f** for oleic acid/oleate vesicles. **a**, **d** In dilute buffer; **b**, **e** in 1.0 wt% PEG20000; **c**, **f** in 10 wt% PEG20000.

Figure 4 shows TEM images of fatty acid vesicles in crowded medium. Vesicles in dilute buffer showed a lower polydispersity with the size of 80 nm (Fig. 4a). With the increase of PEG20000 concentration, the size of the C10 vesicles increased (Fig. 4b, c). The vesicles were connected each other by filaments which was similar with a well-documented transitions of lipid vesicles with pearling instability induced by polymers^14^. Figure 4d–f shows the TEM images of the C18 vesicles. The vesicles in dilute buffer showed remarkably large bi- or multi-layered vesicles with diameter of about 200 or 1000 nm. Meanwhile, the surface of the vesicles turned to be smooth (Fig. 4e). The size of vesicles decreased with the increase of PEG20000 concentration and finally the vesicles turned to be unilamellar (Fig. 4f). The TEM morphologies along with DLS curves proved that crowded medium increased the size of the vesicles of C10 fatty acid but decreased the size of C18 fatty acid vesicles.

The physical properties of vesicles depend on headgroup packing of the amphiphiles, which changed as a function of aggregate diameter (D_h_)^1^. The catalytic capacity of vesicles surpassed that of micelles and depended on aggregate size, which was greater for the small vesicles than for the large vesicles^15^. Kawamuro et al. ^16^ found that small vesicles were 2–5 fold more effective as catalysis in alkaline hydrolysis and thiolysis of *p*-nitrophenyl octanoate. Garcia-Rio et al. ^17^ found that in the reactions involving the nitroso group transfer, the rate constant in the vesicular systems displayed approximately 35 times greater than that in the micellar systems. In this research, the dispersed fatty acids formed different types of aggregates, depending on the ionization degree of the carboxyl group^18^. Figure 1 and Fig. 3a–c shows that the presence of PEG resulted in the increased size of C10 vesicles, which could probably account for the rate decrease of C10 fatty acid formation. On the contrary, the increased rate of C18 fatty acid production could be attributed to the size decrease of C18 vesicles in crowding medium (Figs. 2 and 3d–f). It was speculated that the rate differences arose mainly from a variation in the capacity of the bilayer to solubilize substrates and to dissociate amphiphiles. In the presence of PEG, vesicles formed from C18 fatty acid could be very active “catalysts” and self-reproduced efficiently so that the final population was monodisperse and the hydrolysis was accelerated. For C10 fatty acid, vesicles aggregated to the large particles and therefore failed to offer efficient surface area for catalysis.

#### Catalytic effect of the planted vesicle in the hydrolysis of fatty acid anhydrides

**Fig. 5:**
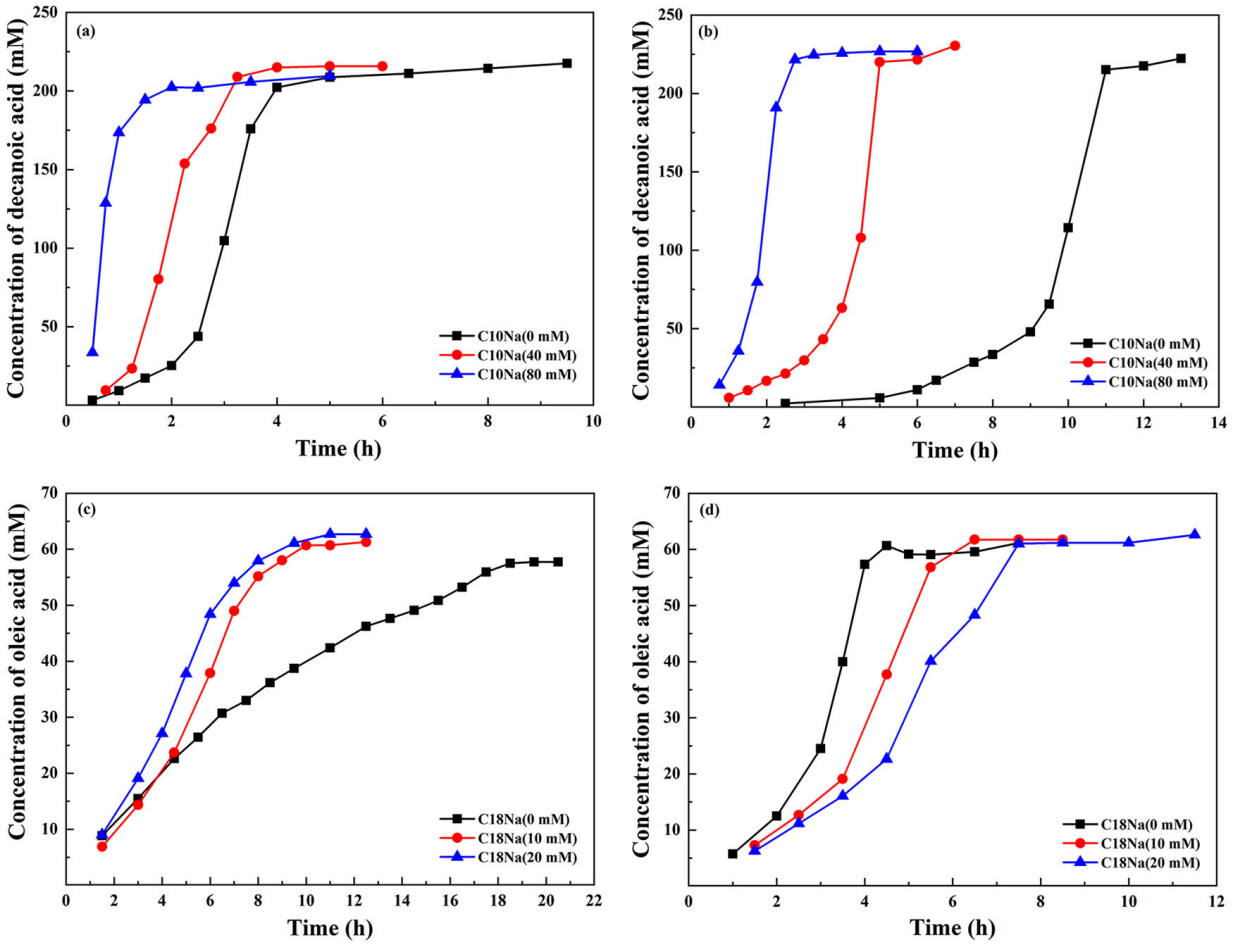
Autocatalytic hydrolysis curves of decanoic anhydride in dilute solution or in PEG2000 with planted vesicles. **a** and **c** in dilute solution; **b** and **d** in PEG2000 (10.0 wt%). **a** and **b** at 60 ^o^C; **c** and **d** at 41 ^o^C.

To investigate further the catalytic effect of vesicles, vesicles were planted before starting the reaction. The preformed vesicles were produced by sodium decanoate or sodium oleate at the corresponding pH. The results were shown in Fig. 5. It can be seen that the rates of C10 fatty acid production in both dilute buffer and PEG2000 were considerably increased in the presence of the planted vesicles. The performance of the planted C18 vesicles in dilute buffer in the hydrolysis of oleic anhydride was similar to that of the above C10 vesicles. However, the introducing of PEG2000 at the same conditions led to the decreased rate of C18 production. These results imply that the mechanism behind crowding-promoted formation of vesicles of C10 or C18 fatty acid was different. In fact, if the total surfactant concentration exceeded the critical vesicle concentration (cvc), the excess amount of surfactant would separate into the “micellar phase”. We found that the fatty acid could form polymer/surfactant aggregates which was inconsistent with PEO-SDS interaction^19^. Though the detailed mechanism was not clear, we speculated that difference in the interaction of polymer surroundings with C10 and C18 fatty acid could not be negligible.

#### Investigation of the viscosity of fatty acids vesicles with or without crowding agents

In order to probe the non-covalence interaction in the system, rheology studies were performed to monitor the viscoelastic changes of fatty acids vesicles with or without crowding agents. As shown in Supplementary Fig. 1, adding a small amount of PEG in fatty acid vesicles solutions profoundly affected the rheological properties of the vesicle solutions. At 60 < γ < 110 s^−1^, the dynamic viscosity of the solutions showed Newtonian behavior. This could be explained by shear-induced deformation and/or aggregate transition of the densely packed vesicles^20^. At γ > 110 s^−1^, however, a shear-thickening behavior of the dilute buffer system was observed. The reason might originate from a structural rearrangement of the polymer under high shear rate. The change in viscosity was related to an expansion of the polymer coil due to surfactant cooperative association and the structure of the fatty acid vesicles. The results in Supplementary Fig. 1 indicated that the shear viscosities of the buffer solution increased when the crowding degree increased. This probably came from the stronger network formation due to macromolecules interchain entangling. Similar results were found for many associating systems^13^.

**Fig. 6:**
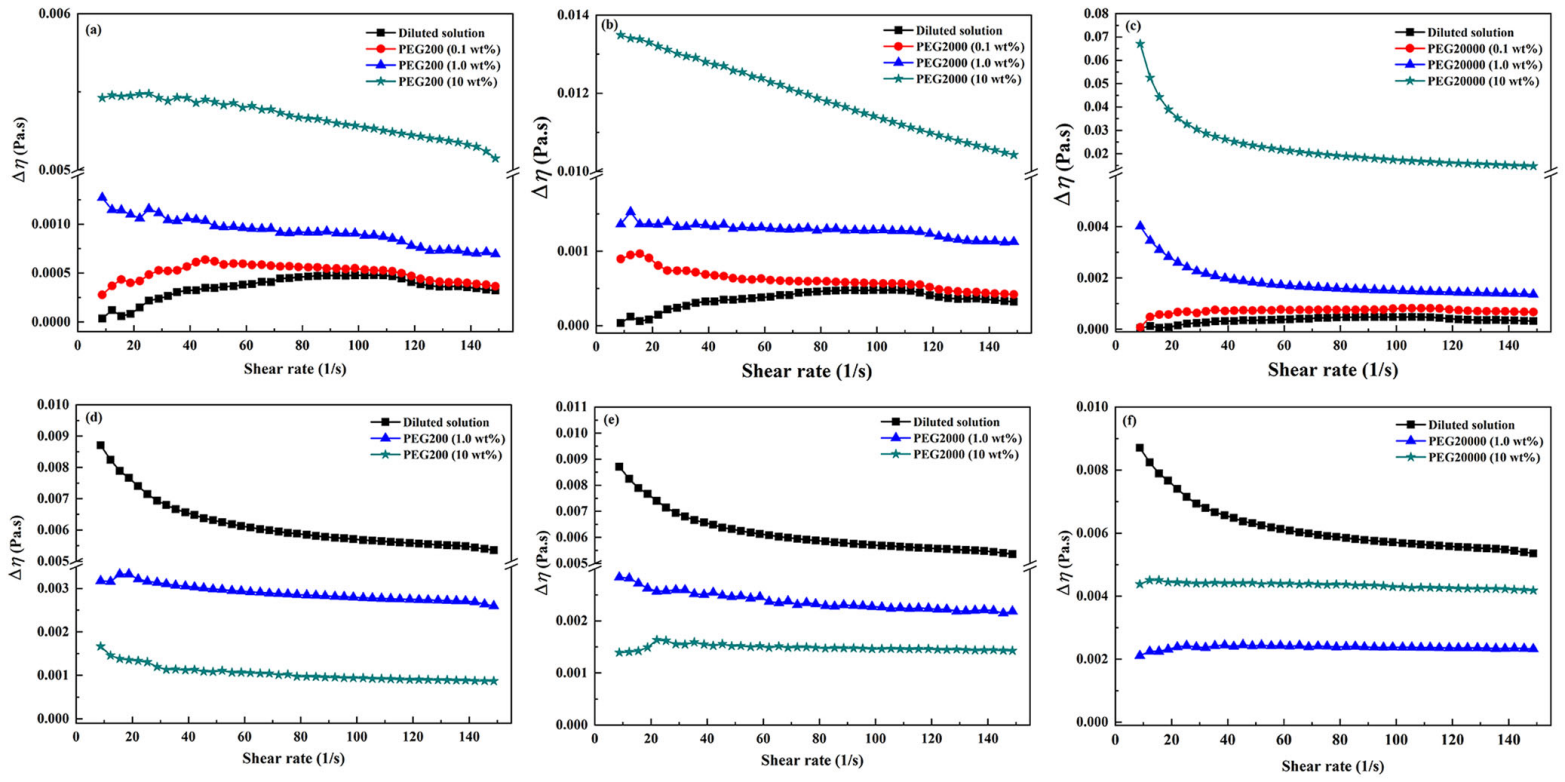
The change of the shear viscosity (Δη) as functions of shear rate for fatty acid vesicles. **a**–**c** For decanoic acid/decanoate vesicles and **d**–**f** for oleic acid/oleate vesicles in dilute buffer and in PEG (the viscosity of medium was subtracted).

Considering that PEG solution alone has a large viscosity, the change of the shear viscosity (Δ*η*) by subtracting the viscosity of PEG as functions of shear rate were obtained and shown in Fig. 6. Δ*η* of C10 vesicles in PEG200, PEG2000 and PEG20000 were larger than that of vesicles without PEG. In addition, Δ*η* increased with the increase of crowding degree. On the contrary, the Δ*η* of C18 vesicles decreased when the crowding degree increased. Liu et al. ^14^. found that the viscosity of vesicles increased when the size of the vesicles increased. We speculate that the increased viscosity of C10 vesicles in crowding was probably related to the size increase of vesicles. The decreased viscosity of C18 vesicles could be attributed to the size decrease. In addition, hydrophobic interaction between the alkyl chains of C18 was stronger than that of C10, therefore smaller vesicles survived in the case of oleic acid. As a result, the C18 vesicles was shrunken in crowding and their hydrodynamic volumes decreased. Anyway, the decreased viscosity induced by crowding at the interface of C18 vesicles facilitated the diffusion, and therefore accelerated the autocatalytic hydrolysis of lipid. Whereas for C10 vesicles, the increased viscosity inhibited the diffusion, which resulted in slowed reaction rate.

#### Oscillation measurements of the crowded network with or without fatty acids vesicles

**Fig. 7:**
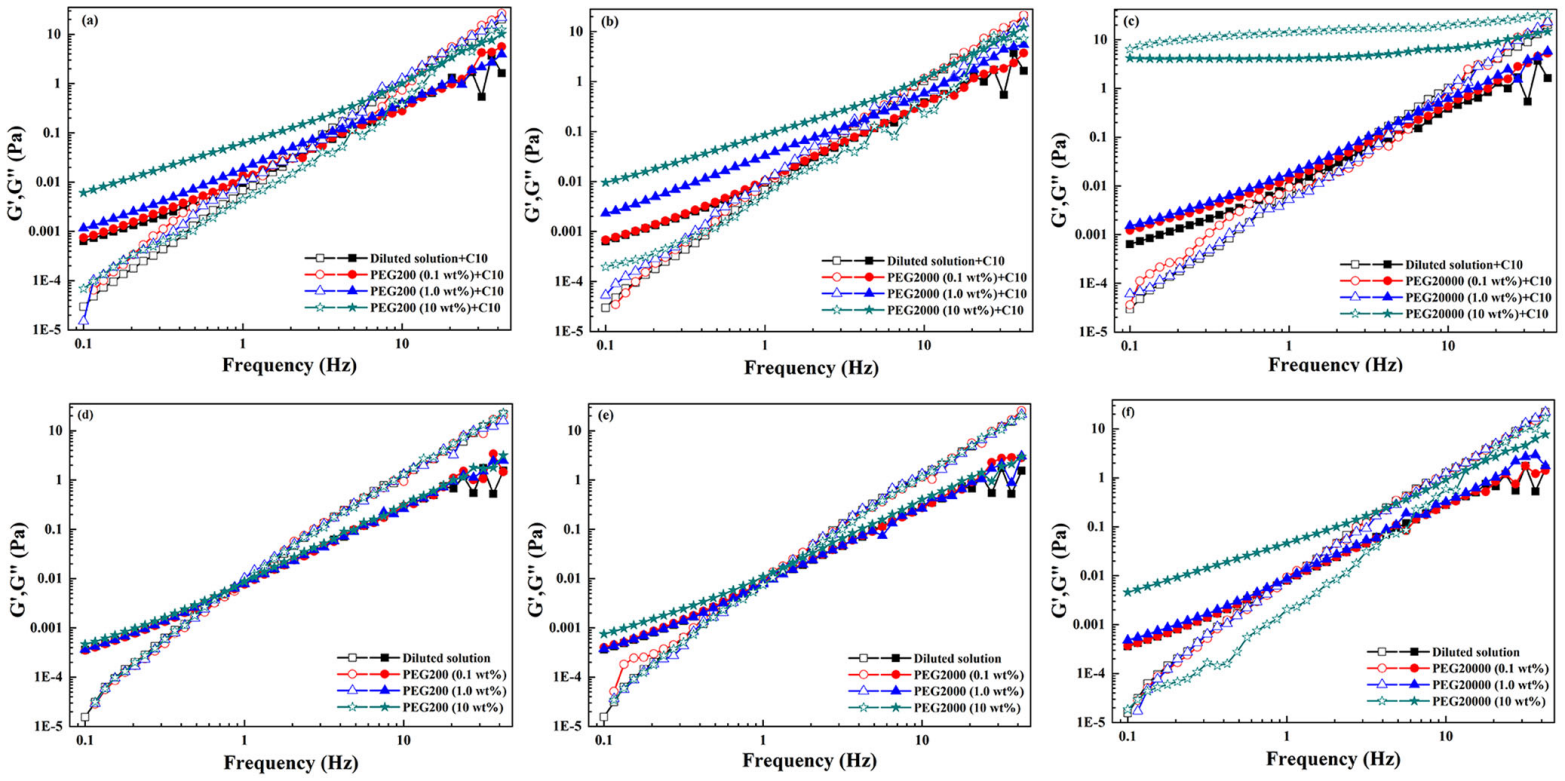
Variation of elastic modulus G' (open symbol) and viscous modulus G" (solid symbol) as a function of frequency for the dilute buffer and PEG solution with or without decanoic acid/decanoate (C10) vesicles. **a**–**c** With decanoic acid/decanoate (C10) vesicles; **d**–**f** without decanoic acid/decanoate (C10) vesicles.

**Fig. 8:**
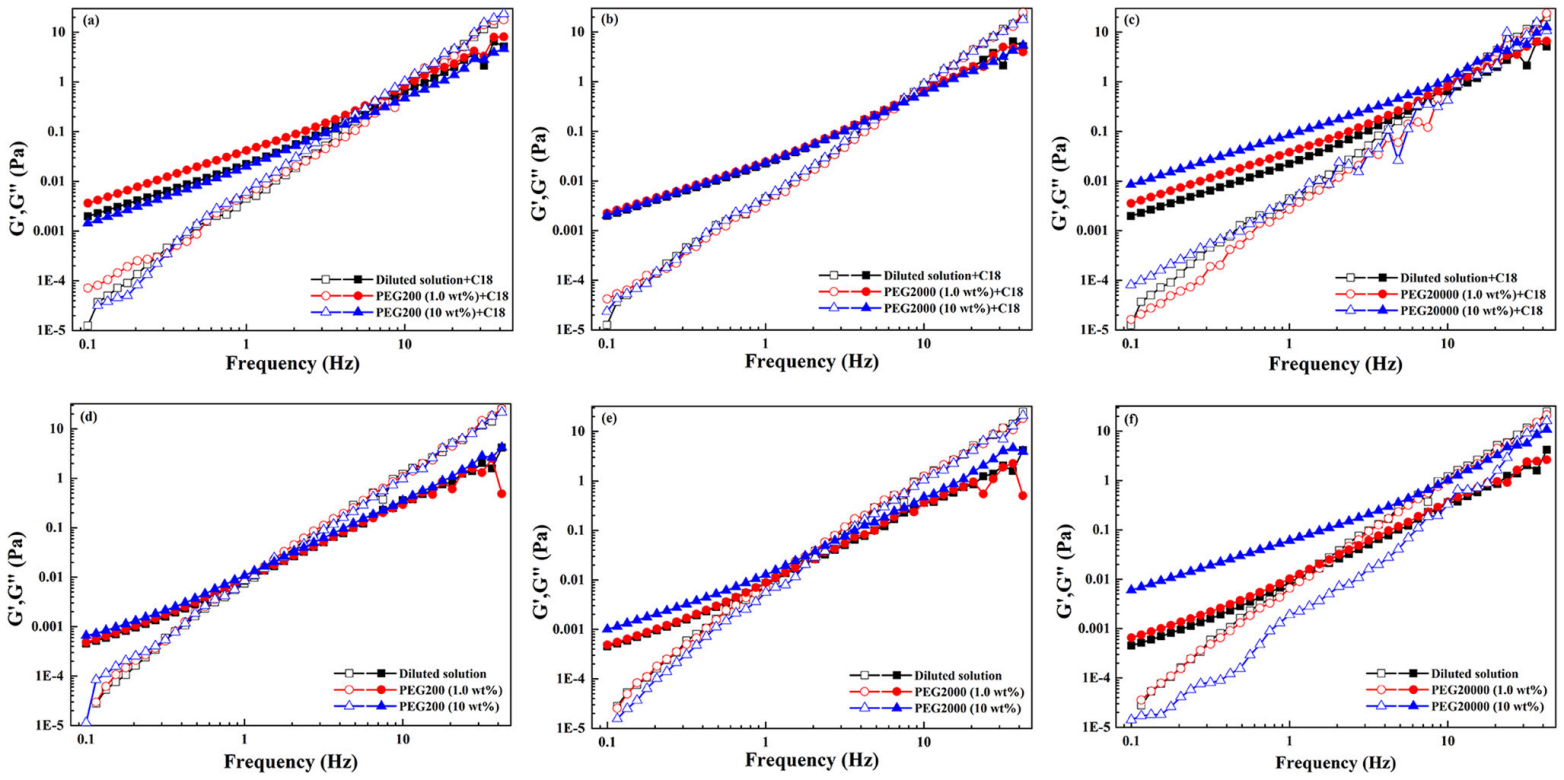
Variation of elastic modulus G' (open symbol) and viscous modulus G" (solid symbol) as a function of frequency (f) for the dilute buffer and PEG solution with or without oleic acid/oleate (C18) vesicles. **a**–**c** With oleic acid/oleate (C18) vesicles; **d**–**f** without oleic acid/oleate (C18) vesicles.

**Fig. 9:**
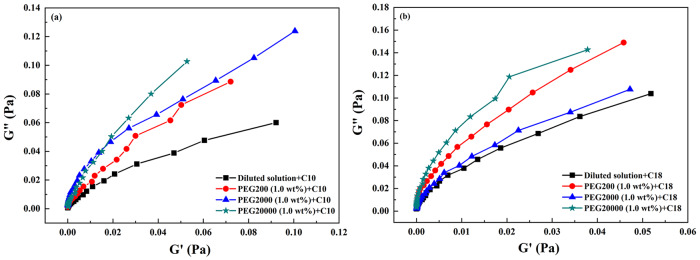
Cole–Cole plots of fatty acid vesicles at low frequency region in dilute buffer and in 1.0 wt% PEG. **a** Decanoic acid/decanoate (C10) vesicles. **b** Oleic acid/oleate (C18) vesicles.

In order to characterize the dynamic properties of macromolecular network with or without fatty acid vesicles, oscillation measurements were performed. The results were shown in Figs. 7 and 8. The elastic modulus G' represented the ability of a deformed PEG coils to “snap back” to its original geometry. The viscous modulus G" represented the tendency of PEG coils to flow under an applied stress. The critical frequency and the critical modulus at the intersection point were denoted as f* and G*, respectively. The reciprocal of the f* corresponded to a relaxation time, τ_R_, which was associated with the elastic energy of network. Both G' and G" increased monotonously for PEG with C10 vesicles (Fig. 7) or PEG with C18 vesicles (Fig. 8) over the entire frequency range. Moreover, G' was smaller than G" at lower frequencies. An intersection occurred for G' and G" curves with the increasing of frequency, after which G' became larger than G", indicating a more pronounced elastic behavior. Compared with PEG alone, f* shifted to higher values, which corresponded to a decreasing critical relaxation time induced by the addition of fatty acid vesicles. When the data were fitted by Maxwell model, a rather poor fit was obtained especially at higher frequencies. As shown in Fig. 9, the cole-cole plots at low frequency region were not semicircular, implying the system was not linear viscoelastic. The PEG-vesicle system did not have single relaxation time and thus the rheological behavior could not be described by Maxwell model^21^. The results showed that whether with or without fatty acid vesicles, G' and G" of PEG were higher in a more crowded medium. However, C10 vesicles in 10 wt% PEG20000 has a dominant G' over the G". G' and G" varied only slightly with the frequency and no cross was observed. All of these are characteristics of the gel, which were closely in packed gel phase^20^.

The changes in f*, G* and τ_R_ of the PEG with or without fatty acid vesicle were listed in Table 1. With the increasing concentration of PEG at the same molecular weight, addition of C10 vesicles shortened the relaxation time but lengthened that of C18 vesicles. The observed lower values of f* in the presence of C18 vesicles implied that the system exhibited more elastic behavior due to a stronger network formation. However, in the presence of C10 vesicles, f* moved to higher values with increasing crowding, indicating that system exhibited more viscous behavior, which was probably due to the collapse of the network induced by the shrink of macromolecules^14^.

**Table 1:** Oscillation measurements of crowded network with or without decanoic acid/decanoate (C10) and oleic acid/oleate (C18) vesicles^a^.

Additive	With C10 vesicles			Without C10 vesicles			With C18 vesicles			Without C18 vesicles		
	f*(Hz)	τ_R_ (s)	G*(Pa)	f* (Hz)	τ_R_ (s)	G*(Pa)	f* (Hz)	τ_R_ (s)	G*(Pa)	f* (Hz)	τ_R_ (s)	G*(Pa)
Buffer	2.075	0.482	0.032	0.923	1.083	0.007	6.670	0.150	0.341	1.334	0.750	0.014
0.1 wt%PEG200+buffer	1.613	0.620	0.024	0.918	1.089	0.007	---			---		
1.0 wt%PEG200+buffer	3.760	0.266	0.109	0.774	1.292	0.005	9.953	0.100	0.820	0.997	1.003	0.009
10 wt%PEG200+buffer	17.32	0.058	2.440	0.853	1.172	0.007	3.655	0.274	0.115	1.390	0.719	0.018
0.1 wt%PEG2000+buffer	1.491	0.671	0.020	0.918	1.089	0.007	---			---		
1.0 wt%PEG2000+buffer	3.376	0.296	0.133	0.725	1.379	0.005	7.870	0.127	0.473	1.148	0.871	0.011
10 wt%PEG2000+buffer	20.80	0.048	3.780	1.594	0.627	0.023	5.606	0.178	0.242	3.010	0.332	0.071
0.1 wt%PEG20000+buffer	6.235	0.160	0.215	0.918	1.089	0.007	---			---		
1.0 wt%PEG20000+buffer	6.445	0.155	0.299	1.184	0.845	0.012	9.810	0.102	0.788	1.954	0.512	0.029
10 wt%PEG20000+buffer	---			13.240	0.076	1.385	28.410	0.035	6.088	26.040	0.038	5.010

^a^The critical frequency (f*), the critical modulus (G*), the relaxation time (τ_R_).

In summary, in a system where fatty acid self-assembly coexisted with lipid hydrolysis, the crowding of the surrounding environment resulted in a reduced production rate of decanoic acid but an increased production of oleic acid. Further exploration revealed that the presence of macromolecules resulted in increased size of C10 vesicles, which could probably account for decreased production rate. On the contrary, the increased rate of oleic acid production could be attributed to decreased vesicles size. Moreover, increased degree of crowding increased the interface viscosity of C10 vesicles but decreased that of C18 vesicles. The decreased viscosity at the interface of oleate vesicles facilitated the diffusion, and therefore accelerated the autocatalytic hydrolysis reaction, whereas decanoate behaved differently. And, C10 vesicles in crowded medium exhibited more viscous behavior, whereas C18 vesicles displayed more elastic behavior. These results suggest that in the presence of crowed medium, formation of fatty acid with shorter hydrophobic chain, diffusion might dominate the reaction; but for long-chain fatty acid, the outcome of macromolecular crowding exhibited in the autocatalytic hydrolysis. Given that the physicochemical mechanisms of digestion and absorption of fatty acids of different chain lengths in the intestine have been less well understood, this research provides useful information for understanding the autocatalyzed production of fatty acid in the presence of self-producing aggregates and macromolecules, which could mimic the physical-chemical processes in lipid digestion.

## Methods

### Materials

Decanoic anhydride (>98.0%), oleic anhydride(>95.0%), sodium decanoate (>99.0%), and sodium oleate (>97.0%) were purchased from TCI-Shanghai. N-[Tris(hydroxymethyl)methyl]glycine (Tricine) (>99.0%), N,N-Bis(2-hydroxyethyl)glycine (Bicine) (>99.0%), polyethylene glycol (PEG, MW = 200, 2000, 20000), and sodium hydroxide(>97.0%) were bought from Aladdin-Shanghai. Isooctane (AR) was from Xilong Chemical. Hydrogen chloride (AR) was purchased from Sinopharm Chemical Reagent Co. Ltd. Double distilled water was used throughout the study.

### Hydrolysis of fatty acid anhydride in crowding

The reaction was performed according to the previous reported procedure^5^. PEG used as crowding agents in desirable concentration. The buffer used in this research were Tricine (0.3 mol/L, pH 8.25), and Bicine (0.2 mol/L, pH 8.50) for hydrolysis of decanoic and oleic anhydride respectively, which were selected according to the literature^22^.

### Determination of the concentration of fatty acid/fatty acid salt during hydrolysis

The total concentration of protonated and depotonated fatty acid in vesicle suspensions was determined by Fourier transform infrared (FT-IR) spectroscopy using a Nicolet DTGS 380 FTIR spectrophotometer with a 0.02 cm CaF_2_ cell over the region of 1900–1600 cm^−1^. The typical procedure could be found in the literature^5^.

### Dynamic light-scattering measurements

The dynamic light scattering (DLS) measurement was determined by a Zetasizer Nano-ZS (Malvern Instruments Ltd., UK) using a laser-Doppler velocimetry technique. A He-Ne laser at a wavelength of 632.8 nm was used and the scattered light at an angle of 173° was detected. Sample solvents were filtered through 0.22 μm Millipore filters before each measurement. Measurements were conducted at 60 ^o^C for decanoic anhydride and 41 ^o^C for oleic anhydride. All measurements were performed in a temperature-controlled chamber. Each test was repeated three or more times.

### Transmission electron microscopy (TEM)

Vesicles were imaged with a JEM-1230 transmission electron microscopy using the negative staining method. A voltage of 120 kV was used for TEM measurements. A drop of vesicle solution was spread on a 200-mesh copper grid coated with a formvar film, and the extra droplet was instantly wiped off by filter paper. After being naturally desiccated, a drop of 1.0% (w/v) uranyl acetate was dripped on the copper grid for about 60 s and the extra droplet was also removed. The grid was dried naturally for about 10 min before TEM observation. Each experiment was repeated two or more times.

### Rheological measurements

Rheological measurement was carried out on a TA DHR-2 rheometer with a Peitier Concentric Cylinder Temperature System. The detailed procedure could be found in the literature^5^. All samples were placed in the temperature-controlled measurement vessel and allowed to equilibrate to the required temperature for at least 20 min prior to conducting measurements.

## Supplementary information

Supplementary information

## Data Availability

Data sharing is not applicable to the main text. The data supporting the findings of this study is available on request from the corresponding authors.
