# Supplementary material for: The effect of macromolecular crowding degree on the self-assembly of fatty acid and lipid hydrolysis
Source: NPJ Sci Food. 2023 Jul 26;7:39. doi: 10.1038/s41538-023-00213-2 (PMC10372097; doi:10.1038/s41538-023-00213-2)
Supplement: Supplementary file 1 — Supplementary information [file 41538_2023_213_MOESM1_ESM.pdf]

Supplementary information

The effect of macromolecular crowding degree on the self-assembly of fatty acid and lipid hydrolysis

Yu-Long Sun[1,2], Bing-Qiang Ge, Mi-Zhuan Li[1], Lei Wang and Zhong-Xiu Chen[1]*

1. Molecular Food Science Laboratory, College of Food & Biology Engineering, Zhejiang Gongshang University, Hangzhou, 310018, China

2. ACON Biotech (Hangzhou) Co., Ltd. Hangzhou, 310018, China

Corresponding author：Zhong-Xiu Chen, email: zhxchen@zjgsu.edu.cn

Address: Molecular Food Science Laboratory, College of Food & Biology Engineering, Zhejiang Gongshang University, Hangzhou, Zhejiang 310035, China.

Tel: 86-571-28008980

Fax: 86-571-28008900

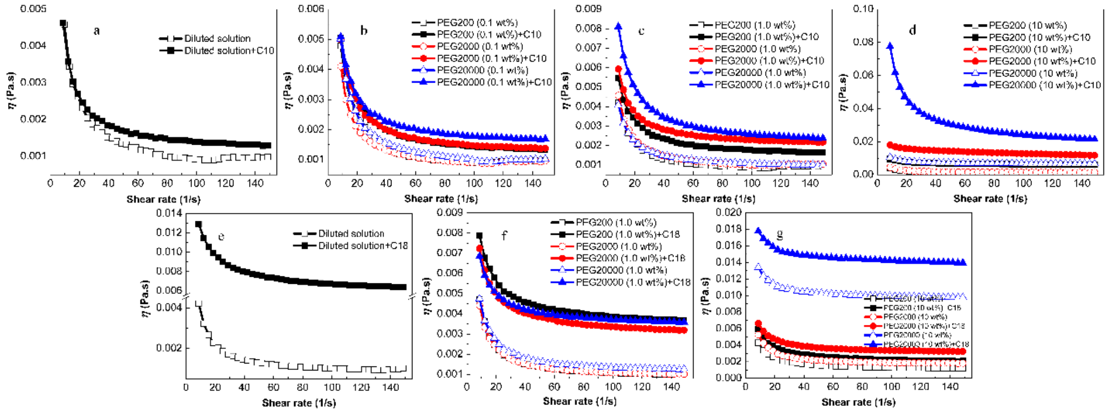

Supplementary Figure 1. Shear viscosity as functions of shear rate for the dilute buffer (a, e) and the PEG solution (b, c, d, f, g) with (solid symbol) or without (open symbol) the decanoic acid/decanoate (a-d) or oleic acid/oleate vesicles (e-g), respectively.
